# Effects of risk exposure on emotional distress among Chinese adults during the COVID-19 pandemic: The moderating role of disruption of life and perceived controllability

**DOI:** 10.3389/fpsyt.2023.1147530

**Published:** 2023-04-26

**Authors:** Xinyan Xiong, Rita Xiaochen Hu, Chuanfang Chen, Wenyuan Ning

**Affiliations:** ^1^School of Sociology, Huazhong University of Science and Technology, Wuhan, P. R. China; ^2^School of Social Work, University of Michigan, Ann Arbor, MI, United States; ^3^Department of Psychology, University of Michigan, Ann Arbor, MI, United States; ^4^School of Marxism, Zhongnan University of Economics and Law, Wuhan, P. R. China

**Keywords:** risk exposure, disruption of life, perceived controllability, emotional distress, the COVID-19 pandemic

## Abstract

**Background:**

COVID-19 affects not only the physical health of individuals but also their mental health and different types of risk exposures are believed to have different effects on individual emotional distress.

**Objective:**

This study explores the relationships between risk exposure, disruption of life, perceived controllability, and emotional distress among Chinese adults during the COVID-19 outbreak.

**Methods:**

This study is based on an online survey conducted during the COVID-19 pandemic, from 1 to 10 February 2020, with a total of 2,993 Chinese respondents recruited through convenience and snowball sampling. Multiple linear regression analysis were used to examine the relationships among risk exposure, disruption of life, perceived controllability, and emotional distress.

**Results:**

This study found that all types of risk exposures were significantly associated with emotional distress. Individuals with neighborhood infection, family member infection/close contact, and self-infection/close contact had higher levels of emotional distress (*B* = 0.551, 95% CI: −0.019, 1.121; *B* = 2.161, 95% CI: 1.067, 3.255; *B* = 3.240, 95% CI: 2.351, 4.129) than those without exposure. The highest levels of emotional distress occurred among individuals experiencing self-infection/close contact, while the lowest levels of emotional distress occurred among individuals experiencing neighborhood infection and the moderate levels of emotional distress occurred among individuals experiencing family member infection (Beta = 0.137; Beta = 0.073; Beta = 0.036). Notably, the disruption of life aggravated the effect of self-infection/close contact on emotional distress and family member infection/close contact on emotional distress (*B* = 0.217, 95% CI: 0.036, 0.398; *B* = 0.205, 95% CI: 0.017, 0.393). More importantly, perceived controllability lowered the strength of the association between self-infection/close contact and emotional distress, as well as family member infection/close contact and emotional distress (*B* = −0.180, 95% CI: −0.362, 0.002; *B* = −0.187, 95% CI: −0.404, 0.030).

**Conclusion:**

These findings shed light on mental health interventions for people exposed to or infected with COVID-19 near the beginning of the pandemic, particularly those who themselves had COVID or had family members with COVID-19 risk exposure, including being infected/having close contact with an infected person. We call for appropriate measures to screen out individuals or families whose lives were, or remain, more severely affected by COVID-19. We advocate providing individuals with material support and online mindfulness-based interventions to help them cope with the after-effects of COVID-19. It is also essential to enhance the public’s perception of controllability with the help of online psychological intervention strategies, such as mindfulness-based stress reduction programs and mindfulness-oriented meditation training programs.

## 1. Introduction

Coronavirus disease-2019 (COVID-19) not only threatens people’s physical health but also has a huge impact on their psychological well-being (1). Emotional distress during the COVID-19 pandemic could be affected by environmental (e.g., media) and individual-level factors.

For one thing, the widespread media attention in the face of this novel virus created an ‘information epidemic’ (2), which might have enhanced individuals’ public health compliance in the COVID-19 pandemic (3) and led to positive attitudes and behavioral changes to respond to the risk (4), but might also have led to some consequences (e.g., lack of individual control) (5). This is because frequent media exposure may amplify individuals’ perceptions of COVID-19 risk and thus lead to negative emotional responses (6). Also, individuals may become information overloaded by frequent exposure to pandemic-related information (2), leading to a sharp increase in short-term stress, which may increase their attention to external stimuli and impair their cognitive responses (7).

For another, after the initial outbreak of COVID-19, 31 provinces in mainland China implemented lockdown measures to control the spread of the outbreak, including home quarantine, closing public places, following social distancing, and working from home online. These sudden changes, along with the fear of infection and the increasing uncertainty and uncontrollability of the epidemic, triggered virus-related stress in individuals (8, 9), which led to further psychological reactions. Consequently, individuals were prone to suffer from various emotional problems, such as panic, anxiety, feelings of emptiness, anger, etc. (10–13). Also, in particular, people with COVID-19 risk exposure tended to have higher levels of anxiety, fear, anger, and other adverse emotional reactions (13–16). Research has shown that emotional distress problems in the population have increased dramatically throughout the COVID-19 pandemic (17).

### 1.1. Risk exposure and emotional distress

As noted by the stress process model, stressors are environments and experiences that are difficult to adjust to. Exposure to stressors can deleteriously affect emotions, cognitions, behavior, etc. (18). The COVID-19 pandemic itself is a stressor that exposes people to the risk of illness and death (19). Therefore, exposure to COVID-19 is a major risk factor for psychological problems. Individuals’ higher levels of direct exposure, or other experiences associated with the pandemic, might have a particularly strong impact on an individual’s psycho-emotional reactions, including suffering from anxiety, fear, depression, and anger. Exposure experiences might include oneself being infected with COVID-19 (16), having close contact with people diagnosed with COVID-19 or showing symptoms of COVID-19 (20), working in high-risk locations or settings (21), the presence of confirmed cases in the community (13, 14), and having family members or friends diagnosed with COVID-19 (15). For instance, studies have shown that individuals who have been in contact with suspected COVID-19 cases or infected objects have higher levels of anxiety symptoms (22). Also, one study showed that people who lost a loved one to COVID-19 were more likely to feel anger than those who did not (23). Another study pointed out that, compared to those people living geographically near, living in, or traveling to Wuhan, those persons who had a direct exposure experience exhibited stronger predictors of depression and other psychological problems (24). Exposure experiences might include being infected, being at high risk of infection, or having a close relationship with an infected individual or an individual at high risk of infection—perhaps a family member, a friend, or a neighbor. Thus, more attention needs to be paid to the impact of these various experiences on individuals’ mental wellbeing, whether the pandemic impacted them personally or a person close to them.

Another study found that people with infected acquaintances had significantly higher levels of depression and also experienced higher levels of anxiety when a family member was infected (25). Almost simultaneously, another study found that higher degrees of anger were experienced by infected individuals, although this anger did not carry over when loved ones were infected (26). Although these studies differentiated between acquaintances and family members on measures, they did not include other types of close social connections, such as neighbors or people one might know who had not been exposed, nor did they compare differences in the strength of the effects of exposure from members of different relational intimacy on individual emotional distress. Therefore, to better understand the relationship between risk exposure and emotional distress during COVID-19, we focus on classifying the direct exposure risk specifically to explore the impact of the exposure risk of different types of members on an individual’s emotional distress and attempt to obtain descriptive results (9) on the differences in the intensity of their effects.

### 1.2. Disruption of life and perceived controllability as moderators

Disruption of life and perceived controllability may also play a moderating role in the relationship between risk exposure and emotional distress. While COVID-19 risk exposure is a greater stressor for individuals, some individuals appear to be more vulnerable to it. As a result, some people suffer more emotional distress despite being exposed to the same level of COVID-19 risk.

#### 1.2.1. Disruption of life as a moderator

##### 1.2.1.1. Disruption of life and emotional distress

In addition to putting people at risk of infection, the COVID-19 pandemic also caused varying degrees of disruption to people’s normal life activities, which led to psychological distress (27). The pandemic exposed people to a range of certain social risks, such as social isolation, economic loss, job insecurity, health insecurity, etc., which increased the uncertainty of life, and could have a serious impact on people’s mental health (27–29). A related study showed that job-related changes caused by COVID-19, such as changes in workload and reductions in income, lead to higher levels of general life dissatisfaction and anxiety among individuals (27). Also, another study indicated that disruptions to daily life were predictors of higher levels of depression, such as poor access to basic supplies (e.g., medicine, food, and toilet paper), having to move unexpectedly, having to cancel a trip or experiencing a major disruption in travel plans, or having to cancel or postpone important events (28). Furthermore, patients with chronic diseases and their family members suffered from higher levels of depression, anxiety, and stress, as healthcare facilities mainly treated COVID-19 patients and public transport was suspended, which significantly affected routine treatment and care for chronic diseases (29, 30). As mentioned above, the pandemic’s disruption of an individual’s life should be considered when examining emotional distress during the COVID-19 pandemic.

##### 1.2.1.2. The moderating role of disruption of life

According to cumulative risk models, exposure to multiple COVID-19 risk factors tends to be associated with more severe mental health outcomes than experiencing a single risk exposure (31), which was supported by empirical research (32). Thus, it can be speculated that cumulative risk sources associated with the COVID-19 pandemic, such as COVID-19 exposure risk and disruption of life, may lead to excessive negative emotions. Therefore, the interaction between risk exposure and life disruption in predicting individual emotional distress should be considered. Furthermore, as noted by the life-change model, the impact of the COVID-19 pandemic on individual lives increased individuals’ emotional vulnerability in general, making it difficult for people to cope with additional, non-COVID trauma exposure, so people whose lives were highly disrupted by the pandemic would suffer more and various risk exposure effects (33). A previous study of victims of workplace bullying showed that exposure to other negative life events besides bullying may increase PTSD symptoms (34), and the same emotional response may be true for individuals affected by COVID-19. In contrast, the facilitator model (33) suggests that life disruption of the pandemic may help people to acquire experiences in coping with stress, which may benefit them in coping with COVID-19 exposure and reduces their psychological distress. That is, the potential role of COVID-19 pandemic disruption to life between individual risk exposure status and emotional distress is unclear, and more empirical evidence is needed. Surprisingly, few current studies on the effects of COVID-19 on mental health discuss the interaction effects of risk exposure and disruption of life.

#### 1.2.2. Perceived controllability as a moderator

##### 1.2.2.1. Perceived controllability and emotional distress

Perceived controllability related to COVID-19 is a situational controllability that refers to the belief in one’s ability to exert influence on the external environment (35, 36). According to Lazarus and Folkman (37), controllability is related to an individual’s evaluation of whether the resources available can help the individual cope with a threat. Individuals who assess more controllability in stressful situations may develop more positive beliefs and behaviors (38, 39), such as optimism, confidence, and protective behavior, leading to better mental health outcomes. Conversely, lower levels of feelings of controllability, such as overgeneralizing the danger of the event and exaggerating the possibility of further catastrophic events, may generate situational fear and lead to lasting stress disorders (40, 41). Previous studies have shown that belief of controllability was linked to public motions and psychological symptoms during public health emergencies. For example, studies of emotional psychological distress during COVID-19 had shown that perceived controllability was a significant negative predictor of depression, anxiety, and stress (42), and conversely, individuals who perceived risk as uncontrollable experienced more depression and posttraumatic stress disorder (9). However, it has also been argued that the adaptive value of controllability does not always work; strong control beliefs in environments with low opportunities for control have instead led to a decline in individuals’ psychological well-being (43). For example, when unemployed victims had higher control beliefs before they lost their jobs, they experienced more distress after job loss (44). Thus, there is no consensus on the relationship between perceived controllability and emotional distress during acutely stressful events, and more evidence is needed.

##### 1.2.2.2. The moderating role of perceived controllability

According to stress and coping theory, stress results from the interaction between the individual and the environment (45). In stressful situations, people make a primary appraisal of the threat and severity of stressors and a secondary appraisal of their coping resources (46). The appraisal is the subjective perception of whether an individual believes an event is controllable. Specifically, applied to the COVID-19 pandemic, the pandemic was recognized as a stressor affecting mental health in all countries (47), which was an objective experience in individuals’ lives; however, individuals could take the initiative to cope with COVID-related stressors in their environments. Therefore, in the face of the comparable risk exposure situation, not all individuals exposed to it may have the same level of stress reaction (18), and individuals with higher perceived controllability were more likely to be less affected by COVID-19 exposure (48).

The self-efficacy mechanism also suggests that the controllability and predictability of risk can help people enhance their sense of self-efficacy (49). Conversely, beliefs of uncontrollability reduce people’s self-efficacy and confidence when exposed to stress, leading to an increase in people’s experience of stress and thus increasing their emotional distress (50, 51). In brief, perceived controllability should be considered as an interactive factor that may reduce individuals’ emotional distress. A previous study has discussed the interaction of COVID-19-related exposure and perceived uncontrollability on psychological outcomes, but researchers have mainly focused on exploring the mediating role of perceived uncontrollability between media exposure (which was indirect exposure) (6) and psychological outcomes, without giving attention to the effect of perceived uncontrollability (or controllability) between direct exposure and psychological outcomes. In addition, insufficient attention has been paid to the moderating role of perceived uncontrollability (or controllability) in the above relationships. Hence, the present study sought to explore the moderating role of perceived controllability between direct risk exposure and emotional distress.

### 1.3. Objectives and hypotheses

**Figure 1:**
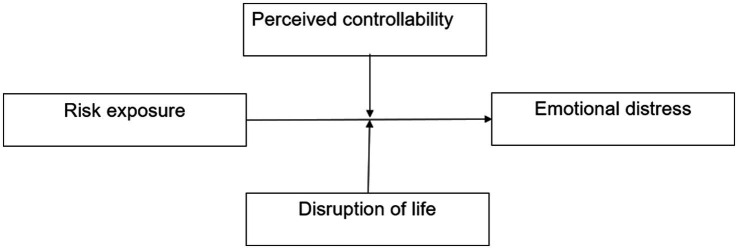
The analytical framework of risk exposure affecting emotional distress.

This study aims to examine the association between types of risk exposures and emotional distress among Chinese adults during the COVID-19 pandemic, utilizing perceptions of the disruption of life and perceived controllability as moderators (see Figure 1).

Correspondingly, we propose the following hypotheses. The first hypothesis (*H1*) focuses on risk exposure. We hypothesize that respondents who had higher levels of COVID-19 risk exposure would have higher emotional distress. Further, we try to propose an exploratory question: different types of risk exposure may be differentially associated with emotional distress in individuals, with neighborhood infection likely to have the weakest association and self-infection/close contact the strongest. The second hypothesis (*H2*) relates to the moderation effect of risk exposure and disruption of life. We hypothesize that the pandemic’s disruption to life may strengthen the relationship between risk exposure and emotional distress. Last, the third hypothesis (*H3*) pertains to the moderating effect of perceived controllability. We hypothesize that perceived controllability would mitigate the impact of risk exposure on emotional distress during the COVID-19 pandemic.

## 2. Materials and methods

### 2.1. Study design

The data for this study were obtained from the Novel Coronavirus Epidemic Psychosocial Survey conducted online^1^ from 1 to 10 February 2020. Convenience sampling and snowball sampling were used to recruit participants. The respondents were Chinese citizens aged 18 or older. The recruitment process consisted of two parts: first, researchers designated several key contacts according to occupation, age, gender, and province; second, the designated contacts were asked to share the survey link to their WeChat groups (an instant messaging software widely used in China). People in the WeChat groups were encouraged to forward the survey link to their WeChat friends. Finally, due to the shortage of medical staff during the outbreak, which may have led to a lag in COVID-19 diagnoses, survey recruits might not have had time to complete the questionnaire. Considering the sample representativeness, a supplementary sample was taken from medical staff. When participants clicked on the survey link, they received informed consent information about confidential negotiations and privacy protection. Recruits could decide to agree and continue to participate or disagree and close the survey. Participants usually completed the questionnaire in 10–20 min and were asked to answer one question before they could move on to the next question until they completed all questions.

The sample size is calculated by the following formula (52): $N=\frac{{\left(Z_{\frac{\alpha }{2}}\right)}^{2}\times p\left(1-p\right)}{d^{2}}$ , where $Z_{\frac{\alpha }{2}}$ is the standard normal variate [at 5% of type 1 error (*p* < 0.05) is 1.96, and researchers usually set $Z_{\frac{\alpha }{2}}$ at 1.96 (53)]; p is the expected prevalence proportion of emotional distress. The study showed that the prevalence of emotional distress such as anxiety and depression in the Chinese population during the COVID-19 pandemic was around 8.3–35.1% and 14.6–48.3% (47), respectively, but to maximize the value of p(1-p) and thus maximize the sample size, we take *p* = 0.5 into the calculation; d is absolute error or precision (when p is between 10 and 90%, d is recommended to be set to 0.05) (53). Based on the above value settings, the minimum sample size for this study was calculated to be 384. In view of the possible invalid responses, we decided to collect a larger sample. Finally, a total of 2,993 respondents in mainland China were recruited, from medical workers, social service providers, teachers, students, the unemployed, farmers, workers, and other occupations.

**Figure 2:**
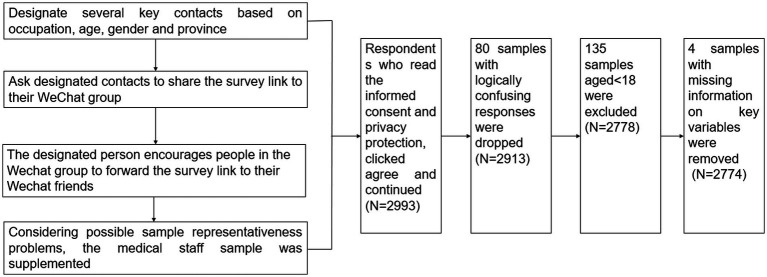
Flow chart of the study design.

Among participants, medical workers working on the front line of the pandemic were at the highest risk of COVID-19 infection. Community workers also had an elevated risk of COVID-19 exposure because they undertook major service tasks such as community group purchasing, food delivery, community screening of potentially infected persons, and assisting in transporting infected persons for medical treatment. Samples younger than 18 years (*n* = 135) or poor quality samples (such as logical confusion, missing information on key variables, etc.) were excluded, and 2,774 samples were finally included in this study (flowchart see Figure 2).

### 2.2. Measurements

#### 2.2.1. Outcome variable

##### 2.2.1.1. Emotional distress

The measurement of emotional distress was adapted from the Discrete Emotions Questionnaire (DEQ) (54), which was measured using four specific emotions: panic, worry, anger, and emptiness, respectively, representing the four dimensions of the original Discrete Emotions Questionnaire. Each emotion was assessed with a 5-point Likert scale ranging from 1 (never) to 5 (all the time). Respondents reported the frequency of each item according to their emotional state over the last month, then summed all items to get a score ranging from 4 to 20. The higher the score, the more severe the respondent’s emotional distress. The Cronbach’s alpha of this scale was 0.776. To assess the fitness of the scale, confirmatory factor analysis was used to explore the validity of the scale in this study, and the results showed that all the items met the measurement requirements (results after linking errors of anger and emptiness according to modification indices: *χ*^2^/*df* = 8.413, *p* < 0.05; RMSEA = 0.052, CFI = 0.998, SRMR = 0.009).

#### 2.2.2. Moderating variables

##### 2.2.2.1. Disruption of life

Disruption of life refers to the extent to which Chinese adults perceived their lives to be disrupted by the COVID-19 pandemic. Based on studies related to the disruption of people’s lives by the COVID-19 pandemic and SARS pandemic (28, 55–57), seven items were used to evaluate the COVID-19 pandemic’s disruption of individuals’ lives, organized under four dimensions: work, finances, daily life, and health. Items examined included overtime work, economic loss, travel changes, being stranded, loss of livelihood, delayed medical care, and lack of supplies. Each item was rated on a 4-point Likert scale ranging from 1 (no impact) to 4 (a very large impact), and the seven items were then added up to get a total score ranging from 7 to 28, with higher scores indicating greater disruption to an individual’s life. This scale showed good internal consistency and test–retest reliability. The Cronbach’s alpha was 0.865 in this study. Confirmatory factor analysis was performed to assess the fit of these seven items, and the results implied that one factor contributed to the correlation structure of these seven underlying item factors (*χ*^2^/*df* = 51.586, *p* < 0.000; RMSEA = 0.135, CFI = 0.914, SRMR = 0.049).

##### 2.2.2.2. Perceived controllability

Referring to research on perceived risk controllability of COVID-19 and SARS (6, 9, 58), five items were used to measure an individual’s perceived controllability of COVID-19, including the controllable degree of society over COVID-19 (uncontrollable to controllable), the avoidable degree of individuals over COVID-19 (avoidable to inevitable), the familiarity with COVID-19 knowledge (familiar to unfamiliar), the possibility of the general public contracting COVID-19 (possible to impossible), and the degree of impact of COVID-19 on society (small to large). We used a seven-point Likert score for each item and reverse-coded all items, with a total score ranging from 5 to 35 when all items were added up. Accordingly, the higher the score, the higher the level of perceived controllability. A confirmatory factor analysis on these three latent variables indicated that the model fit well (results after linking errors of the likelihood of COVID-19 infection in the general population and the impact of COVID-19 on society according to modification indices: *χ*^2^/*df* = 7.501, *p* < 0.000; RMSEA = 0.048, CFI = 0.987, SRMR = 0.019). The Cronbach’s alpha was 0.64 in the current study.

#### 2.2.3. Independent variable

##### 2.2.3.1. Risk exposure

Due to the extreme infectivity and hidden transmission of COVID-19, the exposure risk level of close contact with infected persons was also high. Thus, personal infection status and a history of close contact with an infected person were combined. Then, the final comprehensive risk exposure variable was constructed, which could be divided into four dimensions according to the relational closeness: “no risk exposure, neighborhood infection, family member infection/having close contact with an infected person, and self-infection/having close contact with an infected person” were assigned a score of 1 to 4: the higher the score, the higher the risk exposure level. If participants had any two or more categories of COVID-19 risk exposure among themselves, family members, and neighbors, only the answer with the highest score was used in the analysis. Additionally, risk exposure was a categorical variable in the current study.

#### 2.2.4. Confounding variables

As in previous studies on mental health during COVID-19 (9, 24), the confounding variables in this study mainly included three aspects: demographic characteristics, socioeconomic characteristics, and geographical location variables. Demographic characteristics variables included gender (male, female), age (18–40, 41–60, over 60), educational attainment (middle school or below, high school/technical school, junior college or above), ethnicity (Han, ethnic minorities), marital status (yes, no), and religious beliefs (yes, no). Socioeconomic status variables included job (frontline high-exposure workers, including healthcare workers, aid workers, and community workers; second-line service providers for epidemic prevention and control, including civil servants and social workers; and others), member of the Communist Party of China (CPC) (yes, no), and household income (below average, average, above average). The geographical location variable refers to specific provinces (Hubei Province, other provinces in mainland China).

### 2.3. Statistical analysis

Descriptive analysis was applied to describe the characteristics of the sample. For categorical variables, such as risk exposure, gender, ethnicity, age, member of CPC, religious beliefs, marital status, education, job, geographic location, and household income, frequencies and their percentages were calculated. For continuous variables, such as emotional distress, disruption of life, and risk perception, the mean value, and standard deviation were calculated.

The main analysis was divided into three steps, and the same covariates were used in each step: gender, age, education, ethnicity, marital status, religious beliefs, job, member of CPC, and geographic location. First, since emotional distress was a continuous variable, multiple linear regression models were used to examine the relationship between risk exposure and emotional distress symptoms. Second, based on the above model, the interaction effect of risk exposure and disruption of life on emotional distress was examined by adding the interaction variable (risk exposure × disruption of life). Last, we introduced the interaction variable (risk exposure × perceived controllability) to explore the moderating effect of perceived controllability in the relationship between risk exposure and emotional distress based on the first model. Stata 13.0 was used to conduct all analysis.

## 3. Results

### 3.1. Descriptive analysis

Table 1 presents the descriptive characteristics of the sample. Among the 2,774 adult respondents, almost 96% were Han ethnicity, only a small number of respondents (*n* = 266, 9.59%) had religious beliefs, about a quarter were members of the Communist Party of China (*n* = 703, 25.34%), and female (*n* = 1,483, 53.46%) and married (*n* = 1,168, 60.13%) accounted for more than half. Most respondents were aged 18–40 years old (*n* = 2,169, 78.19%). A small portion of the respondents were workers with high exposure at the frontline of the epidemic (*n* = 455, 16.4%), compared to a lower proportion of civil servants and social workers who indirectly served during the epidemic (*n* = 178, 6.42%), and the remaining respondents were employees almost unrelated to the epidemic (*n* = 2,141, 77.18%). In addition, most respondents considered their household income to be at the average level of the population (*n* = 2,388, 86.09%), while a minority considered it to be above average (*n* = 67, 2.42%). Regarding educational attainment, most of the respondents had a junior college degree or above (*n* = 2,137, 77.04%), and only 9.34% (*n* = 259) had a middle school education or below.

**Table 1:** Descriptive analysis of sample characteristics.

	*N*	%
**Gender**		
Male	1,291	46.54
Female	1,483	53.46
**Ethnicity**		
Han	2,656	95.75
Ethnic minorities	118	4.25
**Age**		
18–40	2,169	78.19
41–60	521	18.78
>60	84	3.03
**Religious beliefs**		
Yes	266	9.59
No	2,508	90.41
**Member of CPC**		
Yes	703	25.34
No	2,071	74.66
**Marital status**		
Yes	1,668	60.13
No	1,106	39.87
**Educational attainment**		
Middle school or below	259	9.34
High school/technical school	378	13.63
Junior college or above	2,137	77.04
**Job**		
Frontline high-exposure workers	455	16.4
Second-line service providers for epidemic	178	6.42
Others	2,141	77.18
**Geographic location**		
Other provinces in mainland China	2,344	84.50
Hubei province	430	15.50
**Household income**		
Below average	319	11.50
Average	2,388	86.09
Above average	67	2.42
**Risk exposure**		
No risk exposure	2,515	90.66
Neighborhood infection	158	5.70
Family member infection/close contact	39	1.41
Self-infection/close contact	62	2.24
	**Mean**	**SD**
Emotional distress	9.16	3.5
Disruption of life	18.62	5.44
perceived controllability	18.98	5.04

SD, standard deviation.

In terms of risk exposure, 90.66% (*n* = 2,515) of the respondents had no risk exposure, 5.7% (*n* = 158) of the respondents reported neighborhood infection, and very few of the respondents had family members who were infected or had close contact with the infected (*n* = 39, 1.41%), with only 2.24% (*n* = 62) of the respondents reporting being infected or having close contact with an infected person. The average scores for disruption of life and perceived controllability among respondents were 18.62 (range 7–28, SD = 5.44) and 18.98 (range 5–35, SD = 5.035) respectively, indicating that the disruption to people’s lives caused by COVID-19 pandemic and people’s perceived controllability were both at medium levels. Regarding the emotional distress variable, the mean value for emotional distress was 9.16 (range 4–20, SD = 3.5). Finally, 430 respondents (15.5%) were from Hubei Province, the worst-hit area in mainland China at that time, and 2,344 respondents (84.5%) were from other provinces in China.

### 3.2. The association between risk exposure and emotional distress

Table 2 presents the multiple linear regression analysis results for the relationship between risk exposure, disruption of life, perceived controllability, and emotional distress among Chinese adults. After controlling for the relevant variables, Model 1 indicated that all types of risk exposures were strongly associated with higher degrees of emotional distress (*B* = 0.551, 95% CI: −0.019, 1.121; *B* = 2.161, 95% CI: 1.067, 3.255; *B* = 3.240, 95% CI: 2.351, 4.129). In detail, compared with unexposed individuals, individuals who were infected or had close contact with infected individuals seemed to have the highest level of emotional distress (Beta = 0.137), followed by individuals with infected family members/close contact (Beta = 0.073), and individuals with neighborhood infection seemed to have the lowest emotional distress (Beta = 0.036). Regarding the COVID-19 disruption of life, model 2 in Table 2 suggested that the COVID-19 disruption of life was strongly related to greater emotional distress (*B* = 0.107, 95%CI: 0.084, 0.129). Finally, model 2 in Table 2 also showed that perceived controllability was negatively significantly associated with emotional distress (*B* = −0.222, 95%CI: −0.246, −0.197).

**Table 2:** Multiple linear regression analysis of the relationship between risk exposure, disruption of life, perceived controllability, and emotional distress.

	Model 1-emotional distress			Model 2-emotional distress		
	*B*	(95%CI)	Beta	*B*	(95%CI)	Beta
**Risk exposure (ref: no risk exposure)**						
Neighborhood infection	**0.551**	(−0.019, 1.121)	0.036*	0.537	(0.014, 1.061)	0.036**
Family member infection/close contact	**2.161**	(1.067, 3.255)	0.073***	1.808	(0.802, 2.814)	0.061***
Self-infection/close contact	**3.240**	(2.351, 4.129)	0.137***	2.345	(1.525, 3.166)	0.099***
Disruption of life				**0.107**	(0.084, 0.129)	0.166***
Perceived controllability				**−0.222**	(−0.246, −0.197)	−0.319***
R-squared	0.045			0.195		
N	2,774			2,774		

*p < 0.1, **p < 0.05, ***p < 0.001. B, the nonstandardized coefficients. Beta, standardized coefficients. 95% confidence intervals in brackets. All confounding variables (gender, ethnicity, age, religious beliefs, member of CPC, marital status, educational attainment, job, geographic location, and household income) were controlled in the above models. The bold numbers indicate statistically significant confidence coefficients.

### 3.3. The moderating effect of disruption of life and perceived controllability

**Figure 3:**
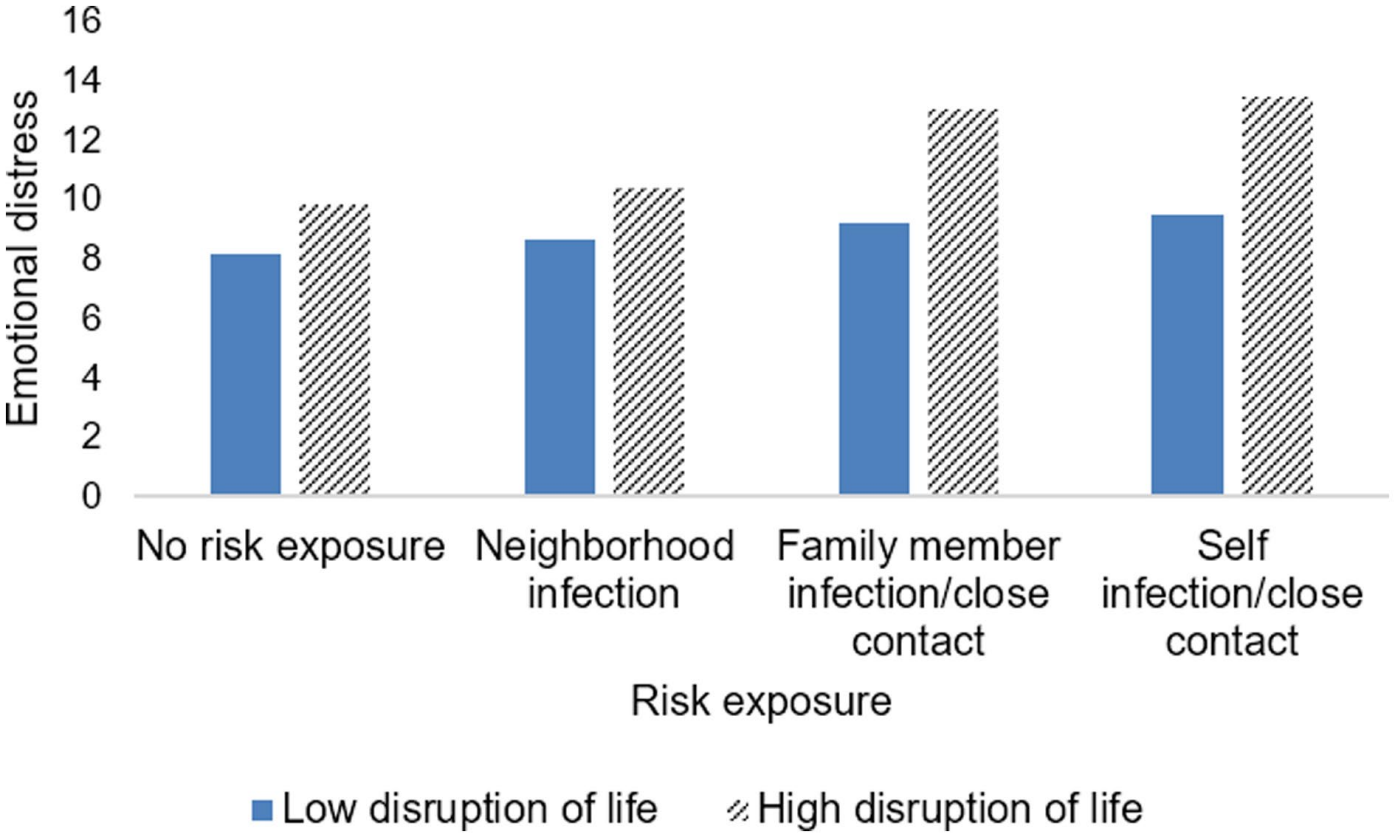
The interaction effect of risk exposure and disruption of life on emotional distress.

**Figure 4:**
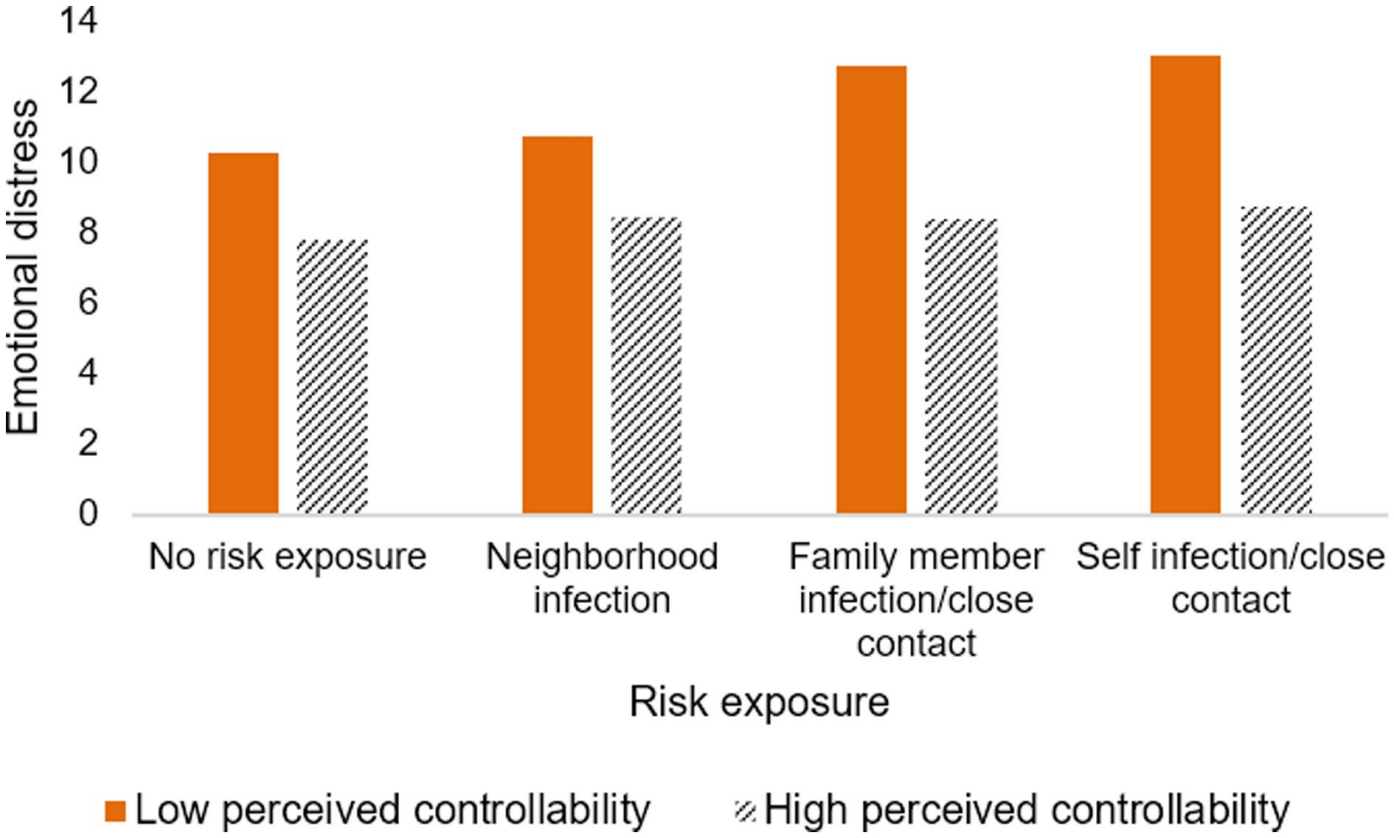
The interaction effect of risk exposure and perceived controllability on emotional distress.

Table 3 and Figures 3, 4 show the interaction effect of risk exposure and disruption of life, as well as risk exposure and perceived controllability on emotional distress. Regarding emotional distress symptoms, models 1 and 2 in Table 3 indicated that both the disruption of life and perceived controllability had a significant interaction effect on the association between self-infection/having close contact and emotional distress and family member infection/having close contact and emotional distress (see below). However, no significant interaction effects of either life disruption or perceived controllability on the relationship between neighborhood infection and emotional distress were observed (see below). Notably, the direction of the moderating effects of life disruption and perceived controllability were different (see below). Specifically, the more an individual’s life was disrupted by the pandemic, the greater the emotional distress for individuals who were infected/having close contact, and the same result for individuals whose family members were infected/having close contact (see Figure 3); that is, disruption of life enhanced the relationship between family member infection/close contact and emotional distress, as well as between self-infection/close contact and emotional distress (*B* = 0.205, 95%CI: 0.017, 0.393; *B* = 0.217, 95%CI: 0.036, 0.398). Furthermore, the interaction effect of self-infection/having close contact and disruption of life (Beta = 0.196) on emotional distress appeared to be stronger than that of family member infection/having close contact and disruption of life (Beta = 0.136). In contrast to the role of disruption of life, the higher the perceived controllability level, the lower the emotional distress of both self-infection/close contact and family member infection/close contact (see Figure 4). Perceived controllability reduced emotional distress in individuals who were infected/closely exposed themselves, or whose family members were infected/closely exposed (*B* = −0.180, 95%CI: −0.362, 0.002; *B* = −0.187, 95%CI: −0.404, 0.030), and the effect of the former seemed to be greater than that of the latter (Beta = −0.123; Beta = −0.115).

**Table 3:** Multiple linear regression analysis for interaction effects of risk exposure and disruption of life on emotional distress and risk exposure and perceived controllability on emotional distress.

	Model 1-emotional distress			Model 2-emotional distress		
	*B*	(95%CI)	Beta	*B*	(95%CI)	Beta
**Risk exposure × disruption of life (ref: no risk exposure)**						
Neighborhood infection × disruption of life	0.010	(−0.094, 0.114)	0.013			
Family member infection/close contact × disruption of life	**0.205**	(0.017, 0.393)	0.136**			
Self-infection/close contact × disruption of life	**0.217**	(0.036, 0.398)	0.196**			
**Risk exposure** × **perceived controllability (ref: no risk exposure)**						
Neighborhood infection × perceived controllability				0.021	(−0.077, 0.119)	0.027
Family member infection/close contact × perceived controllability				**−0.187**	(−0.404, 0.030)	−0.115*
Self-infection/close contact × perceived controllability				**−0.180**	(−0.362, 0.002)	−0.123*
R-squared	0.106			0.172		
N	2,774			2,774		

*p < 0.1, **p < 0.05, ***p < 0.001. B, the nonstandardized coefficients. Beta, standardized coefficients. 95% confidence intervals in brackets. All confounding variables (gender, ethnicity, age, religious beliefs, member of CPC, marital status, educational attainment, job, geographic location, and household income) were controlled in the above models. The bold numbers indicate statistically significant confidence coefficients.

## 4. Discussion

This study investigates the relationship between COVID-19 risk exposure and emotional distress, with disruption of life and perceived controllability as the moderators. Consistent with our hypothesis 1, we found that all types of risk exposure were significantly associated with higher reported emotional distress, controlling for all covariates. In addition, our results showed that both disruption of life and perceived controllability played a moderating role in the link between some types of risk exposures and emotional distress, partially supporting hypotheses 2 and 3 proposed above. Specifically, when exposed to the same level of COVID-19 risk, individuals whose lives were more disrupted by the pandemic might have experienced a higher level of emotional distress symptoms. Meanwhile, our results also suggested that perceived controllability buffered the effects of self-infection/close contact on emotional distress and family member infection/close contact on emotional distress. Overall, our results may provide empirical evidence for mental health intervention strategies applicable during the COVID-19 pandemic, such as digital health technologies, and online intervention techniques based on online mindfulness and meditation (59–61). Utilizing online platforms, these low-cost and easily accessible interventions (61) can benefit individuals who are in emotional distress.

In line with the previous studies (15, 16, 20), risk exposures were associated with higher levels of emotional distress. However, the strength of the associations appeared to vary by the relationship of the exposed to the individual, i.e., emotional distress was highest for self-infection/close contact, followed by family member infection/close contact, and finally, neighbor infection. This may be partly attributed to sociocultural differences and could be explained by individuals’ perceptions of relationship closeness. According to the theory of the “differentiated mode of association” (chaxugeju) (62), Chinese society is organized by concentric relationship circles, with the family as the core and extending outward to relatives, neighbors, friends, strangers, etc. (63). Not all personal relationships are equal, but they are differentiated according to the degree of closeness or distance from the self (64), resulting in different attitudes toward family members, relatives, and friends (63). Compared with Western populations, family is a more important object of social and emotional support for Chinese adults (9, 65). While such affective interaction has been proven to help individuals cope with adverse psychological symptoms caused by the pandemic (9), it could also trigger some negative effects, as talking about traumatic experiences may mean additional trauma exposure in emergencies (66). If someone in the family was infected or at risk of contracting COVID-19, with continuous intimate interaction and emotional sharing, it would bring a sharp increase in the stress psychological response. This is due to the following reasons: individuals inevitably have deep emotional interaction with their relatives, witness the tragic situation of their relatives closely, feel the painful experience, and empathize with their emotions, thus producing “empathy pain” (67). Furthermore, as the stress process model suggests (18), exposure to stressors can trigger a variety of psychological and physiologic responses (68). COVID-19 can be viewed as a stress event that can trigger related negative emotions (23). More importantly, direct exposure to COVID-19 (20), such as a family member contracting COVID-19 (69), can increase the risk of infection in oneself or other family members, leading to more psychological distress in individuals. In the context of the COVID-19 pandemic lockdown, people were advised to stay home to prevent the spread of the virus (70), objectively putting themselves or their family members at potential risk of infection while being relatively less affected by their neighbors. Therefore, the emotional distress of someone in the household who was infected or at high risk of infection was more serious than neighbor’s infection. Last, another explanation could be that the risk of infection within the family may make individuals more stressed, resulting in negative emotions or psychological reactions (71). Given the infectivity and long latency of COVID-19, many people may fear that they could unknowingly become infected and spread it to family members (72). Given the critical role of risk exposure on emotional distress, it is necessary for the government to use Internet-based platforms to provide material support and psychological support to protect the mental health of individuals with COVID-19 risk exposure, especially those who are infected or at high risk of infection themselves, or who have family members at high risk of infection. First, spatial epidemiological analysis methods (mainly combining big data processing techniques such as machine learning and natural language processing with spatiotemporal data analysis) are used to identify key regions and key populations where mental health problems increase over time (73, 74). For example, sentiment content posted on popular social media platforms by users in areas with a high concentration of COVID-19 cases is analyzed to identify and monitor users’ emotional states (73). Second, provide the public with various online psychoeducational interventions such as mindfulness stress reduction, positive meditation, cognitive behavioral therapy, cognitive restructuring, emotional freedom techniques, narrative exposure therapy, and stress management techniques (59, 75); Third, use location or multiple sources of epidemic data (e.g., hospital visit data, population health platform data, government health department data, etc.) to predict epidemic transmission trends, thereby concentrating medical resources in high-risk exposure areas and dynamically visualizing (medical resource mapping) regional medical resources to provide easy healthcare access to individuals with COVID-19 risk exposure (76). Finally, develop a self-service intelligent service system to help people understand COVID-19-related trends and knowledge with the help of AI.

More importantly, the life disruption caused by COVID-19 acted as a moderator between self-infection/close contact and emotional distress, and between family member infection/close contact and emotional distress. Specifically, the disruption of life potentially exacerbated emotional distress in individuals who were infected/had close contact with infected persons or whose family members were infected/had close contact with infected persons, and the emotional distress appeared to be greater in the former than in the latter. This could be understood within the framework of the cumulative risk model (31), which implies that experiencing multiple stressors associated with COVID-19 may lead to worse psycho-emotional responses. Life disruptions caused by the pandemic, such as school suspensions and unemployment, had led to anxiety and other negative psychological outcomes (27, 77). Moreover, previous research has also established that someone in the family (perhaps an individual him- or herself or a family member) infected or at high risk of infection (e.g., having close contact) tended to have more negative emotions (23, 24). According to the cumulative risk model (31), these negative emotions would be more severe for those whose lives were disrupted by COVID-19. In addition, another explanation may be that the life disruptions of COVID-19, such as livelihood shocks and job losses, reduced the potential resilience of households or individuals to other stresses beyond their control (78). For example, respondents with reduced household income were forced to save less, leading to greater vulnerability to future shocks, such as health risks from pandemics (78). The last explanation related to exposure bias, whereby individuals whose lives were vulnerable to shocks, such as suffering unemployment and being stranded outside the home, were inherently more exposed to risk. A previous study showed that immigrants stranded at the North American border because of travel restrictions had a higher risk of contracting COVID-19 (79), while concerns about health risks were associated with immigrants’ distress and fear (80). Given the above, we believe that public health interventions that screen out people whose lives are most affected by COVID-19 and provide them with material support measures such as livelihood assistance and online psychoeducational interventions including mindfulness techniques and meditation techniques (59) are critical for mental health protection during the pandemic.

Moreover, this study found that perceived controllability mitigated the effects of self-infection/close contact on emotional distress and family member infection/close contact on emotional distress, with perceived controllability appearing to have a stronger effect on the former than the latter. As noted by the stress and coping model (37), COVID-19-related emotional distress was the result of the interaction between objective real risk and individual subjective appraisal, and perceived controllability is a key secondary appraisal factor in the stress-coping process. According to the compensatory effects hypothesis in the risk-resilience model (81), some protective factors can buffer the negative emotional responses to adversity, and these include both internal and external resources. Perceived controllability was considered a positive psychological resource that implies individual self-efficacy and optimistic belief in the face of threat (82) and was a protective factor that not only buffered the effects of the COVID-19 pandemic on adverse psychological outcomes but also promoted psychological well-being (e.g., life satisfaction) during the pandemic (48, 83). Perceived controllability, as a positive psychological belief, acts similarly to confidence in society: individuals who perceive COVID-19 risk to be at a controllable level may hold higher expectations of themselves and society as being able to cope with the threat of COVID-19 (51), thereby reducing the anxiety and stress associated with infection or possible infection. Also, another explanation may relate to the fact that risk controllability predicts higher perceived effectiveness of social distance and more preventive behaviors (38). That is, individuals with high perceived controllability may have a more fact-based understanding of COVID-19. Therefore, when they or their family members are infected or at risk of infection, they may adopt more active coping styles, such as maintaining safe social distancing at home, wearing masks, washing hands frequently, and communicating online, which not only ensures the close connection between family members but also reduces the psychological stress of being infected or potentially infecting their family members. In summary, we should learn from these results and improve the public’s perceived controllability through public health education and psychological interventions, such as effective communication with the public, online psychological interventions (including the use of cognitive–behavioral techniques, Mindfulness-Based Stress Reduction and meditation techniques group training programs) (59–61), and guide the public to improve controllability in stressful situations.

### 4.1. Limitations and implications

This study has several limitations. First, the cross-sectional study design makes it difficult to accurately identify causality in the relationship between risk exposure and individual emotional distress outcomes. Future studies can use experimental designs and longitudinal data to further clarify causal relationships. Second, network sampling technology was convenient and safe during the pandemic but also causes sample selection bias. Some groups with relatively poor internet access (such as low-educated people, the elderly, and rural people) were excluded, making the relationship between risk exposure and emotional distress inappropriately assessed due to the differences in risk exposure among the population. Previous studies showed that people with low education were at higher risk of exposure to COVID-19 due to their occupation (84). As the number of COVID-19 infections in urban areas was much higher than that in rural areas, the risk exposure of rural populations was overestimated. Third, we could not show the trend of the relationship between risk exposure and emotional distress symptoms over time. Our data were collected during the early stages of the COVID-19 outbreak, when a vaccine was not yet available to protect against the virus in all countries. As time goes by, scientific research has not only led to the clinical use of the COVID-19 vaccine in most countries, but also an increasingly comprehensive understanding of COVID-19 among the general public, so that the impact of risk exposure on individual emotional distress may not be as strong as in the early years. Thus, it is prudent to consider potential time changes in interpreting our findings, especially in the post-epidemic era after mass vaccination with the COVID-19 vaccine; Also, future studies could examine changes in this relationship over time, particularly now that the COVID-19 vaccine has been mass vaccinated in most countries, and make longitudinal comparisons with findings from the earliest days of the COVID-19 outbreak. Fourth, our data were collected at the beginning of the COVID-19 pandemic, when most countries (85, 86) were experimenting with various lockdown strategies and policies to control the spread of the pandemic as much as possible in the face of this novel virus. Therefore, our data do not measure perceptions of media information or government policies. For that reason, it would be best to cautiously generalize the findings of this research and consider potential changes in policy or societal perceptions related to COVID-19 over time. As countries around the world shift their policies toward the COVID-19 pandemic, future research could consider incorporating these macroenvironmental factors and comparing findings with studies from the beginning of the pandemic to understand how emotional distress changes over time and circumstances during the pandemic. Finally, the generalization of the research conclusions is best placed in a similar geographical and cultural context. Our sample was collected in mainland China, influenced by the collective culture prevalent in East Asia, where people’s subjective understanding of risk exposure may differ from that in western countries, resulting in different levels of emotional distress. Future studies can complement comparative studies from the perspective of cultural differences.

Notwithstanding the above limitations, this study still has some important implications. First, we found that risk exposure strongly predicts higher levels of emotional distress, especially among individuals who were infected/had close contact and whose family members were infected/had close contact, suggesting that targeted psychoeducational interventions should be more family oriented. Furthermore, the study showed that the life disruption of COVID-19 enhances the relationship between self-infection/having close contact and emotional distress, as well as the relationship between family members’ infection/having close contact and emotional distress. Therefore, the government should adopt appropriate measures (such as questionnaires and telephone contacts) to identify families or individuals whose lives are greatly affected by the COVID-19 pandemic and provide them with effective support. Third, given the buffering effect of perceived controllability between risk exposure and emotional distress, the government should implement appropriate mental health interventions to enhance individuals’ perceived controllability and reduce their pandemic-related stress. For example, the government provides systematic psychological care for individuals, including online video courses or apps such as Mindfulness-Based Stress Reduction training (MBSR) and meditation exercises, to promote individuals’ mental flexibility skills, thereby improving their subjective perceptions of risk and alleviating their emotions (60).

## 5. Conclusion

This study indicates that individuals with self-infection/close contact, family member infection/ close contact, and neighborhood infection have higher degrees of emotional distress than individuals without risk exposure, and that the level of emotional distress seems to decrease progressively among these four risk exposures. Moreover, the disruption of life by COVID-19 is found to enhance the effects of emotional distress in individuals who were infected themselves/were in close contact with an infected person or had a family member infected/family members in close contact with an infected person. Additionally, perceived controllability buffers the impact of the effects of self-infection/close contact on emotional distress and family infection/close contact on emotional distress. These findings provide important implications for the government in crisis management of similar epidemics in the future; that is, they should not only prevent the spread of epidemics scientifically but also pay attention to the mental health problems of the public and provide them with online health interventions (e.g., mindfulness-based stress reduction programs and mindfulness meditation programs) (59) using videoconferencing, websites, or mobile apps to guide them to effectively adjust their negative emotional reactions.

## Data availability statement

The original contributions presented in the study are included in the article/supplementary materials, further inquiries can be directed to the corresponding author.

## Ethics statement

The Ethics Committee of Huazhong University of Science and Technology reviewed and approved this study. The participants provided their written informed consent to participate in this study.

## Author contributions

XX designed the study and drafted the manuscript. RH revised the manuscript and edited the language. WN and CC revised and proofread the final manuscript. All authors participated in the revision of the manuscript and approved the final version.

## Funding

Funding for this study came from the COVID-19 Pandemic Social Risk Prevention and Control Research Project (2020HZZK032).

## Conflict of interest

The authors declare that the research was conducted in the absence of any commercial or financial relationships that could be construed as a potential conflict of interest.

## Publisher’s note

All claims expressed in this article are solely those of the authors and do not necessarily represent those of their affiliated organizations, or those of the publisher, the editors and the reviewers. Any product that may be evaluated in this article, or claim that may be made by its manufacturer, is not guaranteed or endorsed by the publisher.

## Footnotes

^1^https://www.wjx.cn/app/survey.aspx
